# Serum proteomic predicts effectiveness and reveals potential biomarkers for complications in liver transplant patients

**DOI:** 10.18632/aging.103381

**Published:** 2020-06-12

**Authors:** Wenjing Wang, Bo Wang, Chang Liu, Jing Yan, Xiaofan Xiong, Xiaofei Wang, Juan Yang, Bo Guo, Chen Huang

**Affiliations:** 1 Department of Hepatobiliary Surgery, The First Affiliated Hospital of Xi’an Jiaotong University, Xi’an 710061, P R China; 2 Department of Cell Biology and Genetics, School of Basic Medical Sciences, Xi'an Jiaotong University, Health Science Center, Shaanxi, Xi'an 710061, P R China; 3 Department of Clinical Medicine, Medical College of Yan’an University, Yan’an 716000, P R China; 4 Institute of Genetics and Developmental Biology, Translational Medicine Institute, Xi'an Jiaotong University, Xi'an 710061, P R China; 5 Key Laboratory of Environment and Genes Related to Diseases (Xi'an Jiaotong University), Ministry of Education of China, Xi'an 710061, P R China; 6 Key Laboratory of Shaanxi Province for Craniofacial Precision Medicine Research, College of Stomatology, Xi'an Jiaotong University, Xi'an 710004, P R China

**Keywords:** proteomic, serum biomarker, acute rejection (AR), ischemic-type biliary lesion (ITBL), liver transplantation

## Abstract

Sophisticated postoperative complications limit the long-term clinical success of liver transplantation. Hence, early identification of biomarkers is essential for graft and patient survival. High-throughput serum proteomics technologies provide an opportunity to identify diagnostic and prognostic biomarkers. This study is aimed to identify serum diagnosis biomarkers for complications and monitor effectiveness. Serum samples from 10 paired pre- and post-liver transplant patients, 10 acute rejection (AR) patients, 9 ischemic-type biliary lesion (ITBL) patients, and 10 healthy controls were screened using matrix-assisted laser desorption/ionization time-of-flight mass spectrometry (MALDI-TOF MS) to explore divergence in polypeptide. Then, we used ELISA and western blot analysis to validate the expression of these potential biomarkers, and studied the correlation of proteomic profiles with clinical parameters. ACLY, FGA, and APOA1 were significantly lower in pre-operative patients compared with healthy controls, and these patients had modest recovery after transplantation. Downregulation of both, ACLY and FGA, was also observed in AR and ITBL patients. Furthermore, bioinformatics analysis was performed and the results suggested that the identified proteins were involved in glucolipid metabolism and the clotting cascade. Together, these findings suggest that ACLY, FGA, and APOA1 could be novel non-invasive and early biomarkers to detect complications and predict effectiveness of liver transplantation.

## INTRODUCTION

In recent decades, liver transplantation has become the most effective curative therapy for patients with end-stage liver disease (ESLD). Combined with improved immunosuppressive therapy, surgical techniques, and perioperative management, the 1-year patient survival rate has exceeded 80%. However, transplant recipients still face high morbidity due to complications during the immediate post-transplant period, including rejection, infection and biliary and vascular complications, with the incidence rate ranging from 14% to 55% [1]. For example, the incidence of acute rejection (AR), a thorny clinical problem with lack of timely diagnosis and personalized immune system evaluation, is still as high as 40% [2]. Despite increased interest in the discovery of potential biomarkers for AR in various solid transplantations, few are routinely used in clinical practice. In addition, ischemic-type biliary lesion (ITBL), the most devastating biliary complication, generally along with frequent infection, repeated invasive biliary procedures, and poor prognosis [3], remains a common and elusive complication due to lack of tools for early diagnosis. AR and ITBL are major causes of morbidity, graft loss, and even mortality following liver transplantation. Due to the non-specific signs and symptoms of these diseases, the routine laboratory tests (ultrasound and radiological imaging) have limited value in evaluating graft liver dysfunction. The final diagnosis of these complications still depends on invasive and time-consuming techniques, such as biopsy and cholangiography. Therefore, a non-invasive and rapid diagnosis method is urgently needed to improve the diagnosis, prognosis, and decrease mortality.

The rapid development of serum proteomics has enabled identification of differential changes underlying pathophysiological conditions and therapeutic responses, and uncover potential diagnostic markers in many diseases [4]. Matrix-assisted laser desorption/ionization time-of-flight mass spectrometry (MALDI-TOF MS) has been widely used in protein analysis for rapid and accurate identification of biomarkers and mechanisms [5]. While various cancer biomarkers have been explored [6], only a few studies have focused on non-cancer diseases [7–10] and a little progress is reported on biomarker discovery in solid organ transplantations [11–15]. Proteomics provides a reliable and effective tool to identify disease markers that can help to evaluate the illness state of patients in liver transplantation.

**Figure 1:**
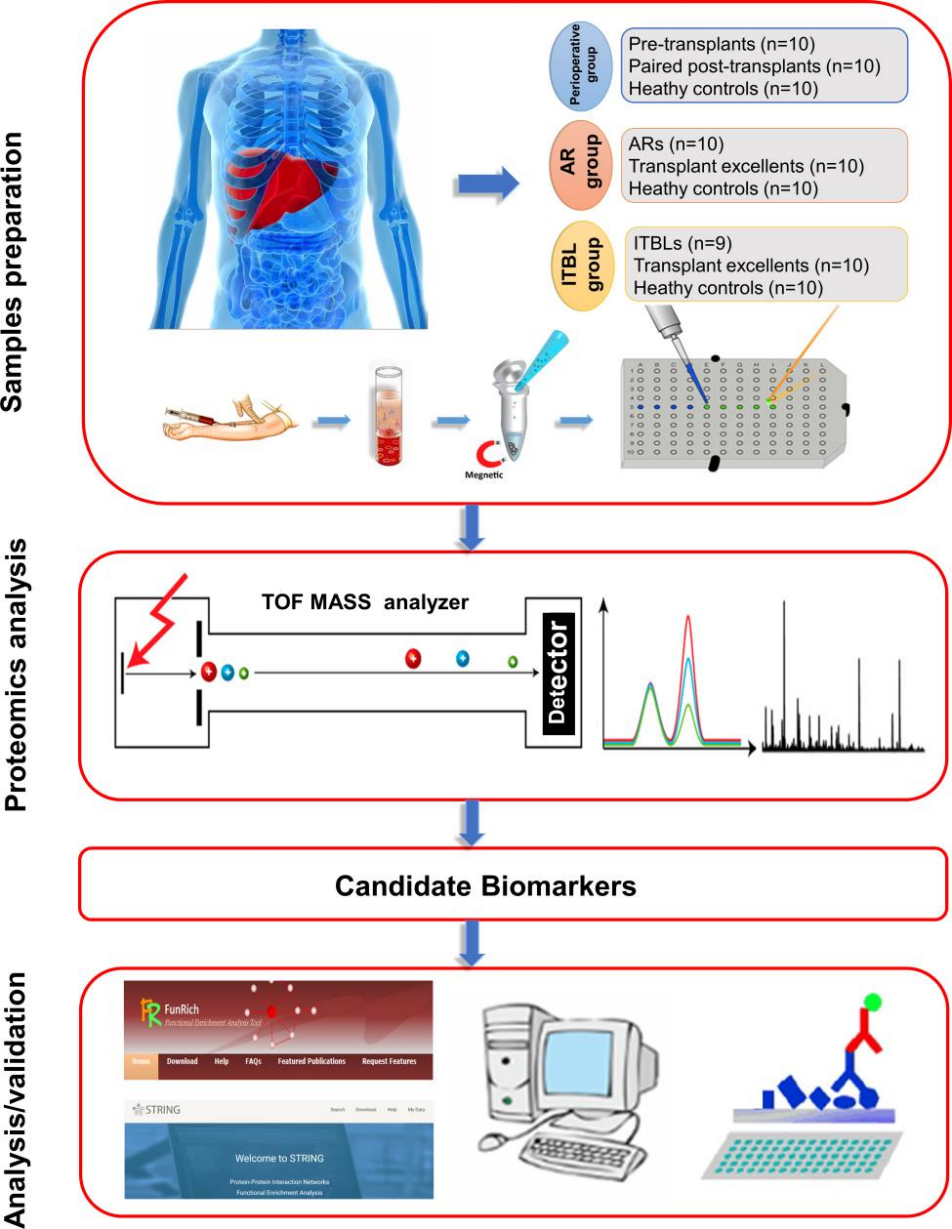
**Schematic illustration of the workflow: from blood collection to analysis and validation.**

In the present study, the serum samples from paired pre- and post-transplant patients, AR patients, ITBL patients, and healthy controls were analyzed by magnetic bead-based weak cation exchange (MB-WCX) purification and MALDI-TOF MS and significant differentially-expressed peaks were selected. ATP citrate lyase (ACLY), fibrinogen alpha chain (FGA) and apolipoprotein A1(APOA1) were identified as potential biomarkers by sequencing and bioinformatic analysis. Finally, the positive correlation between these three biomarkers and clinical information was evaluated, and their exact expressions were validated by enzyme-linked immunosorbent assay (ELISA) and western blot analysis (Figure 1). Herein, we identified ACLY, FGA and APOA1 as serum biomarkers for the diagnosis of complications related to liver transplantation for the first time. Our results demonstrate the potential value of these proteins in assessing the effectiveness of liver transplantation.

## RESULTS

### General information

General information on the donors and recipients evaluated during the perioperative period is summarized in Supplementary Table 1. There were 7 males and 3 females in the pre- and post-operative study group with an average age of 47.88 ± 8.49 years. For patients with AR (8 males and 2 females) and ITBL (7 males and 2 females) complications, the average age was 46.57 ± 8.75 and 52.00 ± 9.60 years, respectively. In healthy controls, there were 7 males and 3 females donors with an average age of 47.25 ± 8.73 years. There was no statistically significant difference between transplant patients and healthy controls with respect to the distribution of age and sex (*p* > 0.05).

In patients with ITBL, the average age of donors was higher than that of transplant excellent patients (55.75 ± 10.01 versus 42.54 ± 17.47 years, *p* = 0.2339), and anhepatic phase was significantly longer than transplant excellent patients (57.60 ± 7.28 versus 45.88 ± 8.43 minutes, *p* = 0.0377). The warm ischemia time was longer in patients with AR than in healthy transplant patients (14.57 ± 7.28 versus 9.63 ± 0.86 minutes, *p* = 0.0996). There were no major differences regarding the other factors.

### Serum proteomic profiles of healthy controls, paired post-transplant patients and pre-transplant patients

**Figure 2:**
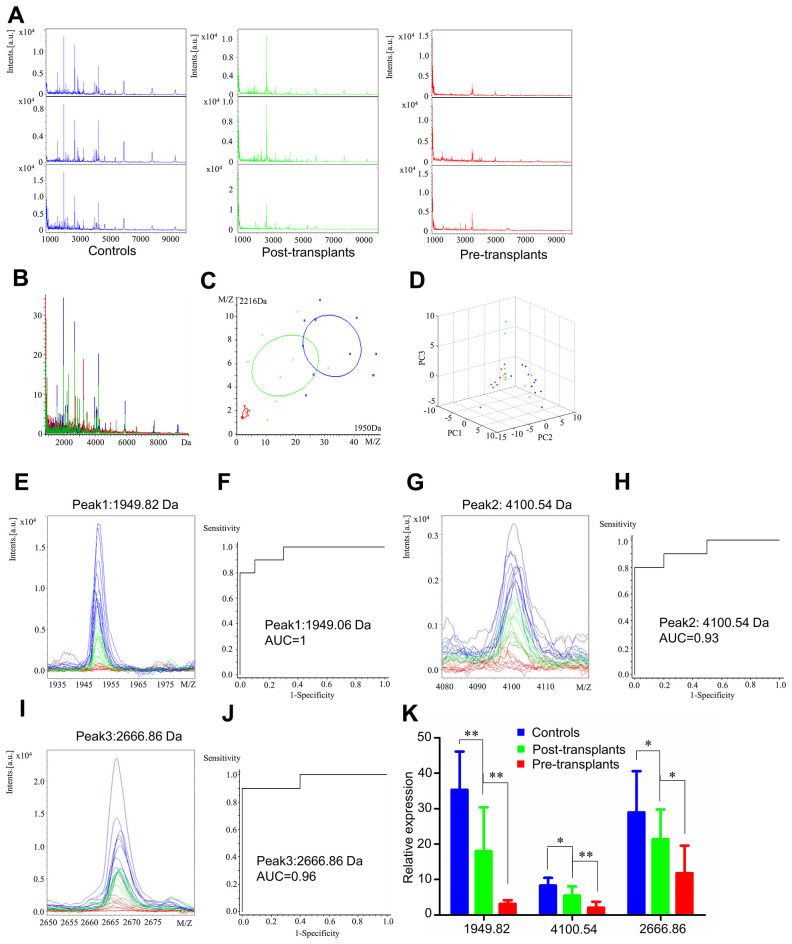
**Serum proteomic profiling analysis for perioperative group, healthy controls (blue), paired post-transplant patients (green) and pre-transplant patients (red).** (**A**) Representative mass spectra of three samples in healthy controls, post-transplant patients and pre-transplant patients in the mass range from 1000 to 10,000 Da. (**B**) Overall sum of the spectra in the mass range from 1000 to 10,000 Da obtained from perioperative group described above. (**C**) Bivariate plot of perioperative group with the most differentiated two peaks (m/z: 2216, 1950). (**D**) 3D plot of perioperative group. (**E**, **G**, **I**) Comparison of the spectra of three peaks in healthy controls, post-transplant patients and pre-transplant patients. (**F**, **H**, **J**) ROC curves for three selected peaks with their AUC values. (**K**) Average expression levels of three selected peaks in healthy controls, post-transplant patients and pre-transplant patients and their respective p-values. Values are expressed as mean ± SD. (***p < 0.001, **p < 0.01, *p < 0.05).

We analyzed mass spectra of samples from healthy controls, paired post-transplant patients, and pre-transplant patients (perioperative group). Spectra are presented for the mass range of 1 to 10 kDa. The mass spectra differed between the healthy controls, paired post-transplant patients, and the pre-transplant patients (Figure 2A and 2B). Principal component analysis revealed that the perioperative group (Figure 2C and 2D) showed a slightly overlapping region, which suggested the possibility of exploring prognostic serum biomarkers to separate pre-transplant patients from control subjects. To evaluate the effect of transplantation, we analyzed the serum proteomic profiles in the perioperative group. ClinProTools analysis identified 79 different peaks, of which 5 differed significantly within the groups (fold change > 1.5; *p* < 0.001). Peaks 1-5 were downregulated in pre-transplant patients compared with healthy controls (Supplementary Table 2). The three most significantly differential peaks (Peak 1, m/z: 1949.82; Peak 2, m/z: 4100.54; Peak 3, m/z: 2666.86) observed in the mass spectra, showed similar values to those of post-transplant patients when compared with the healthy controls (Supplementary Table 2), which suggested that these three peptides could be potential biomarkers for transplant effectiveness.

Comparison of the spectra of these three peaks and their receiver operating characteristic (ROC) curves and area under the curve (AUC) values are displayed in Figure 2. After transplantation, these three peaks showed a tendency to return to levels as in healthy control (Figure 2E, 2G, and 2I). The AUC values of peaks 1, 2, and 3 were 1 (Peak 1, m/z: 1949.06), 0.93 (Peak 2, m/z: 4100.54), and 0.96 (Peak 3, m/z: 2666.86), respectively (Figure 2F, 2H, and 2J). Comparison of the spectra among healthy controls, paired post-transplant, and pre-transplant patients and their mean expression levels are shown in Figure 2K.

### Serum proteomic profiles of healthy controls, transplant excellent patients and AR patients

**Figure 3:**
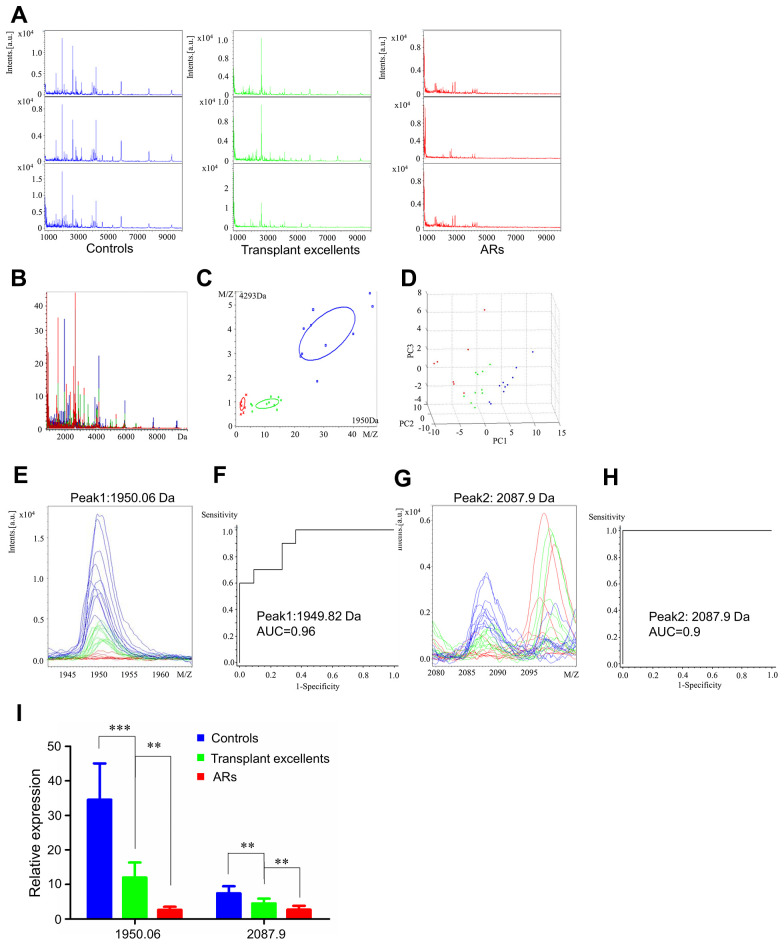
**Serum proteomic profiling analysis for AR group, healthy controls (blue), transplant excellent patients (green) and AR patients (red).** (**A**) Representative mass spectra of three samples in healthy controls, transplant excellent patients and AR patients in the mass range from 1000 to 10,000 Da. (**B**) Overall sum of the spectra in the mass range from 1000 to 10,000 Da obtained from AR group described above. (**C**) Bivariate plot of AR group with the most differentiated two peaks (m/z: 4293, 1950). (**D**) 3D plot of AR group. (**E**, **G**) Comparison of the spectra of two peaks in healthy controls, transplant excellent patients and AR patients. (**F**, **H**) ROC curves for two selected peaks with their AUC values. (**I**) Average expression levels of two selected peaks in healthy controls, transplant excellent patients and AR patients and their respective p-values. Values are expressed as mean ± SD. (***p < 0.001, **p < 0.01, *p < 0.05).

We analyzed serum proteomic profiles in the AR group (healthy controls, transplant excellent patients and AR patients). Representative mass spectra of three samples and overall sum of the spectra differed in healthy controls, transplant excellent patients, and AR patients in the mass range from 1 to 10 kDa (Figure 3A and 3B). Figure 3C and 3D showed little overlapping region in principal component analysis for the AR group. ClinProTools analysis revealed 80 different peaks, of which two peaks (Peak 1, m/z: 1950.06; Peak 2, m/z: 2087.9) differed significantly among the AR group (fold change > 1.5; *p* < 0.001). Peaks 1 and 2 were significantly downregulated when comparing AR patients and transplant excellent patients to healthy controls (Supplementary Table 3).

Peptide mass spectra comparisons of the two peaks (Figure 3E and 3G) were consistent with the numerical results in Supplementary Table 3. The AUC values of Peak 1 (m/z: 1950.06) and Peak 2 (m/z: 2087.9) were 0.96 and 0.9, respectively (Figure 3F and 3H). The relative expression levels of these peptide peaks between healthy controls, excellent patients and AR patients are shown in Figure 3I. These results suggest that expression of two peptides in AR patients was higher than in the control subjects, indicating that they could be candidate diagnostic serum biomarkers for AR.

### Serum proteomic profiles of healthy controls, transplant excellent patients and ITBL patients

**Figure 4:**
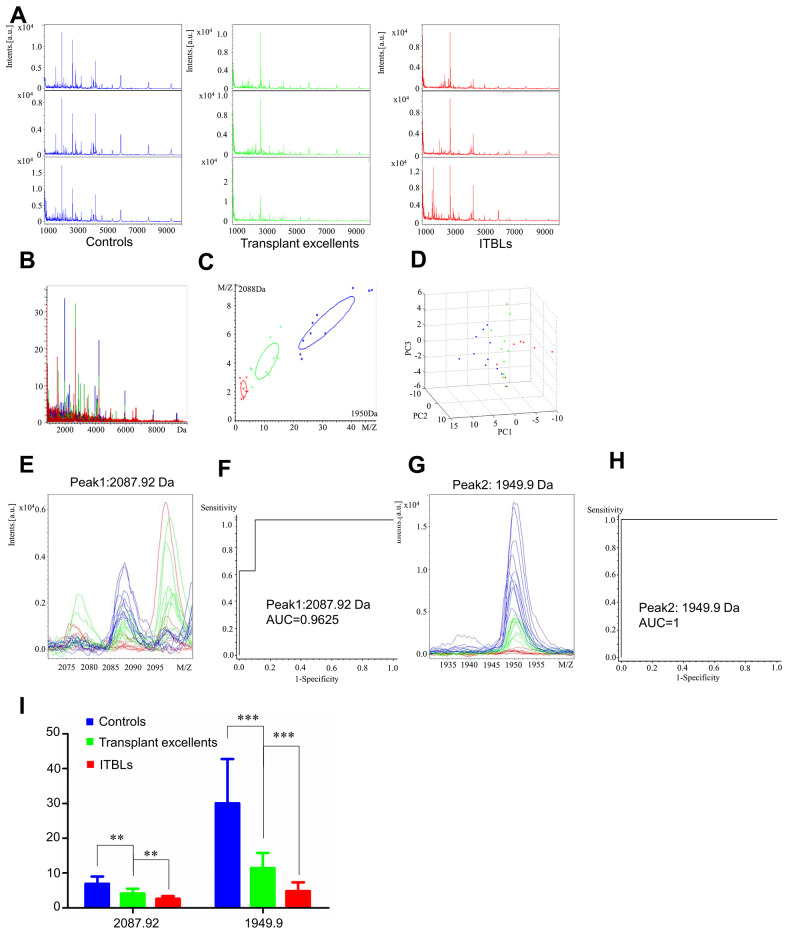
**Serum proteomic profiling analysis for ITBL group, healthy controls (blue), transplant excellent patients (green) and ITBL patients (red).** (**A**) Representative mass spectra of three samples in healthy controls, transplant excellent patients and ITBL patients in the mass range from 1000 to 10,000 Da. (**B**) Overall sum of the spectra in the mass range from 1000 to 10,000 Da obtained from ITBL group described above. (**C**) Bivariate plot of ITBL group with the most differentiated two peaks (m/z: 2088, 1950). (**D**) 3D plot of ITBL group. (**E**, **G**) Comparison of the spectra of two peaks in healthy controls, transplant excellent patients and ITBL patients. (**F**, **H**) ROC curves for two selected peaks with their AUC values. (**I**) Average expression levels of two selected peaks in healthy controls, transplant excellent patients and ITBL patients and their respective p-values. Values are expressed as mean ± SD. (***p < 0.001, **p < 0.01, *p < 0.05).

We also analyzed serum proteomic profiling in the ITBL group (healthy controls, transplant excellent patients, and ITBL patients). Mass spectra differed in healthy controls, transplant excellent patients and ITBL patients (Figure 4A and 4B). Figure 4C and 4D show a slight overlapping region in principal component analysis in ITBL group.

ClinProTools analysis showed 72 different peaks, of which two (Peak 1, m/z: 2087.92; Peak 2, m/z: 1949.9) significantly differed between the three subject groups (fold change > 1.5; *p* < 0.001). Peaks 1 and 2 showed significant downregulation when comparing ITBL patients and transplant excellent patients to healthy controls (Supplementary Table 4).

Peptide mass spectra comparisons of the two peaks (Figure 4E and 4G) were consistent with the numerical results in Supplementary Table 4. The AUC values of Peak 1 (m/z: 2087.92) and Peak 2 (m/z: 1949.9) were 0.9625 and 1, respectively (Figure 4F and 4H). The relative expression levels of these peptide peaks among healthy controls, excellent patients and ITBL patients are shown in Figure 4I. Collectively, these results suggest that these peptides could be potential biomarkers for ITBL.

### Identification of serum peptide biomarkers

All seven peptide peaks mentioned above from the perioperative group (Peak 1, m/z: 1949.82; Peak 2, m/z: 4100.54; Peak 3, m/z: 2666.86), AR group (Peak 1, m/z: 1950.06; Peak 2, m/z: 2087.9) and ITBL group (Peak 1, m/z: 2087.92, Peak 2, m/z: 1949.99) were identified using LC-ESI-MS/MS and the Uniprot database. The MS/MS spectrum of these peptides identified proteins including ACLY (m/z:1949.9), APOA1(m/z: 4100.81) and FGA (m/z: 2087.71 and 2666.86) (Table 1). The sequences of the peptides identified can be found in Supplementary Figure 1.

**Table 1:** Sequence identification of 7 differentially expressed peaks among paired pre- and post-transplant patients, AR patients, ITBL patients and healthy controls.

**Peak**	**m/z**	**Uniprot ID**	**Peptide sequence**	**Identified protein**
**1**	2087.71	P02671	Y.KMADEAGSEADHEGTHSTKRGHAKSRPV.R	Isoform 1 of Fibrinogen alpha chain precursor (FGA)
**2**	4100.81	P02647	Q.DEPPQSPWDRVKDLATVYVDVLKDSGRDYVSQFEGS.A	Apolipoprotein A-I precursor (APOA1)
**3**	1949.9	P53396	K.ILIIGGSIANFTNVAATFK.G	ATP citrate lyase (ACLY)
**4**	2666.86	P02671	A.DEAGSEADHEGTHSTKRGHAKSRPV.R	Isoform 1 of Fibrinogen alpha chain precursor (FGA)

### FunRich functional enrichment analysis and STRING interaction analysis

**Figure 5:**
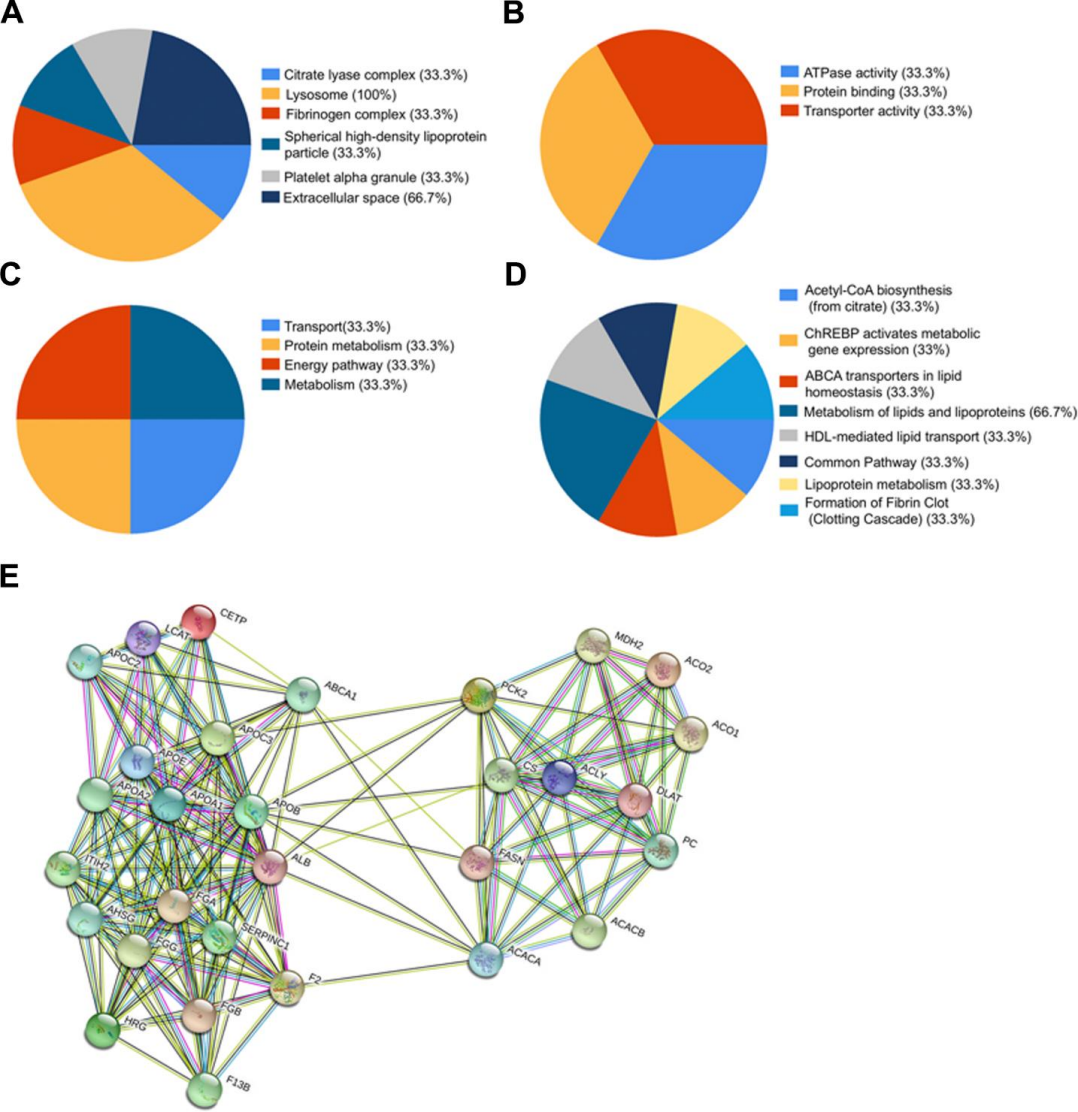
**Bioinformatics analysis of identified proteins.** (**A**–**D**) Funrich analysis of identified proteins. (**A**) Distribution of cellular components of identified proteins. (**B**) Distribution of molecular functions of identified proteins. (**C**) Distribution of biological processes of identified proteins. (**D**) Distribution of biological pathway of the identified proteins. (**E**) Interaction network between identified proteins and their function-related proteins based on prediction results of STRING.

To further investigate the potential function and role of the three proteins, we performed FunRich analysis, and the results are shown in Figure 5A–5D. Firstly, the cellular component ontology described the subcellular structures and macromolecular complexes, and could therefore be used to annotate cellular locations of gene products. The identified proteins were scattered among various cellular components, including the lysosome (100%), extracellular space (66.7%), citrate lyase complex (33.3%), fibrinogen complex (33.3%), spherical high-density lipoprotein particle (33.3%), and platelet alpha granules (33.3%) (Figure 5A). Secondly, the molecular function analysis described the major functions of identified protein patterns, such as ATPase activity (33.3%), protein binding (33.3%), and transporter activity (33.3%) (Figure 5B). Thirdly, the three proteins were involved in a wide range of biological processes, including transport (33.3%), protein metabolism (33.3%), energy pathway (33.3%), and metabolism (33.3%) (Figure 5C). Finally, the biological pathway analysis demonstrated that these proteins were mainly involved in metabolism of lipids and lipoproteins (66.7%), acetyl-CoA biosynthesis (from citrate) (33.3%), ChREBP activates metabolic gene expression (33%), ABCA transporters in lipid homeostasis (33.3%), HDL-mediated lipid transport (33.3%), common pathway (33.3%), lipoprotein metabolism (33.3%), and formation of fibrin clots (clotting cascade) (33.3%) (Figure 5D). Overall, these proteins are very likely to play major roles in the metabolism of lipids and lipoproteins, pyruvate metabolism, and clotting cascade.

To explore the alteration of the protein interaction network in liver transplant patients from different groups, the STRING database (http://string-db.org) was used to generate a network from three seed proteins with their predicted functional partners. The results revealed a strong protein-protein interaction network among ACLY, FGA, and APOA1 (Figure 5E).

### Full protein expression validation of identified peptides in patients’ serum

**Figure 6:**
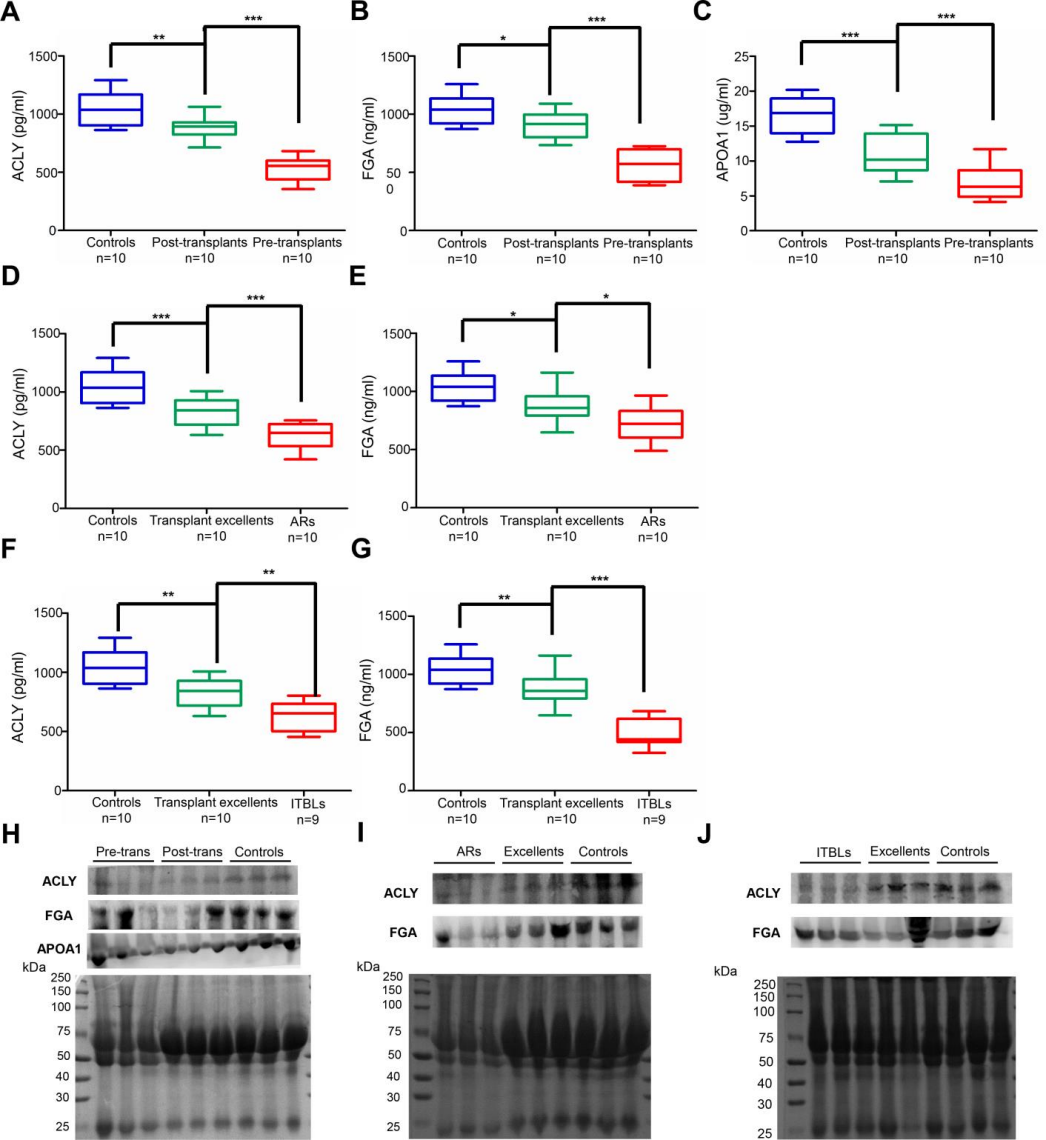
**Validated expression of potential serum biomarkers by ELISA and western blot.** (**A**–**G**) ELISA analysis of ACLY, FGA and APOA1 in the serum of different groups. (**A**–**C**) ACLY, FGA and APOA1 expression in perioperative group. (d-e) ACLY and FGA expression in AR group. (**F**–**G**) ACLY and FGA expression in ITBL group. (***p < 0.001, **p < 0.01, * *p* < 0.05) (**H**–**J**) Confirmation of proteomic results by western blot in the serum of different groups. (**H**) Detection of ACLY, FGA and APOA1 in perioperative group, randomly chose three serum samples from pre-transplants, post-transplants and healthy controls, respectively. (**I**) Detection of ACLY and FGA in AR group, randomly chose three serum samples from ARs, transplant excellents and healthy controls, respectively. (**J**) Detection of ACLY and FGA in ITBL group, randomly chose three serum samples from ITBLs, transplant excellents and healthy controls, respectively. Transferred proteins to the PVDF membrane were detected by Ponceau S stain as western blot loading control.

The potential biomarkers of transplant prognosis and complications diagnosis identified in our study, ACLY, FGA, and APOA1, were further validated with quantitative ELISA using the same cohort of samples (Figure 6A–6G). In the perioperative group, the mean serum concentration of ACLY was 1049.29 ± 150.42 pg/ml (range 863.21-1291.79 pg/ml) in healthy controls, 878.87 ± 96.26 pg/ml (range 713.21-1063.21 pg/ml) in paired post-transplant patients, and 533.50 ± 98.13 pg/ml (range 356.07-681.50 pg/ml) in pre-transplant patients (Figure 6A). The mean serum concentration of FGA was 1042.22 ± 126.63 ng/ml (range 872.50.21-1258.61 ng/ml) in healthy controls, 909.50 ± 115.95 ng/ml (range 734.55-1091.17 ng/ml) in paired post-transplant patients, and 564.39.50 ± 123.92 ng/ml (range 389.41-725.98 ng/ml) in pre-transplant patients (Figure 6B). The mean serum concentration of APOA1 was 16.70 ± 2.63 μg/ml (range 12.77-20.19 μg/ml) in healthy controls, 11.03 ± 2.85 μg/ml (range 7.08-15.13 μg/ml) in paired post-transplant patients, and 6.84 ± 2.38 μg/ml (range 4.14-11.70 μg/ml) in pre-transplant patients (Figure 6C). The expression levels of these three proteins were significantly lower in pre-transplant patients, with all *p* values < 0.0001, showing the same tendency as their peptides (Table 2, Figure 2).

**Table 2:** Correlation analysis between serum potential biomarkers and clinical information in perioperative group.

	**ACLY**		**FGA**		**APOA1**	
**Parameters**	**r**	**P Value**	**r**	**P Value**	**r**	**P Value**
**WBC**	0.123	0.734	0.147	0.684	-0.086	0.814
**RBC**	0.363	0.303	0.267	0.455	0.300	0.399
**PLT**	0.520	0.123	0.442	0.201	0.306	0.390
**NEU**	-0.412	0.236	-0.396	0.257	-0.501	0.14
**LYM**	0.606	0.063	0.634	0.049	0.465	0.176
**MONO**	0.370	0.293	0.416	0.232	0.207	0.567
**ALT**	-0.637	0.048*	-0.430	0.215	-0.391	0.263
**AST**	-0.650	0.042*	-0.445	0.197	-0.403	0.249
**GGT**	-0.485	0.156	-0.434	0.210	-0.518	0.125
**CHO**	0.954	0.000**	0.945	0.000**	0.852	0.002*
**ALB**	0.848	0.002**	0.763	0.01*	0.797	0.006**
**TBIL**	0.032	0.931	0.062	0.865	0.000	0.999
**PT**	-0.586	0.075	-0.590	0.073	-0.501	0.141
**FIB**	0.594	0.07	0.650	0.042*	0.794	0.006**

(*** indicates P < 0.001, ** indicates P < 0.01, * indicates P < 0.05).

In addition, the concentration of ACLY and FGA in healthy controls, transplant excellent patients, and AR and ITBL patients also showed a noticeably gradual decrease (Figure 6D–6G). Concentrations of ACLY in AR patients (629.50 ± 107.90) were significantly lower than those of transplant excellent patients (837.21 ± 124.72, *p* < 0.001), and those in transplant excellent patients were significantly lower than those in healthy controls (1049.29 ± 150.42, *p* < 0.001) (Figure 6D). Concentrations of FGA in AR patients (722.17 ± 148.64) were significantly lower than those of transplant excellent patients (880.83 ± 137.76, *p* < 0.05), and those in transplant excellent patients were significantly lower than those in healthy controls (1042.22 ± 126.63, *p* < 0.05) (Figure 6E). A similar trend was also observed in the peptide analysis of the ITBL group. Expression levels of ACLY and FGA in the ITBL patients were significantly lower than those in transplant excellent patients (*p* < 0.01, *p* < 0.001, respectively) and healthy controls (*p* < 0.001, both) (Figure 6F–6G).

All results indicated that ACLY, FGA, and APOA1 were all expressed at significantly lower levels in pre-transplant, AR, and ITBL patients compared to the other subjects, showing the same tendency as the proteomics. Finally, western blot analysis was performed to verify the relative serum expression changes for the identified proteins in different groups (Figure 6H–6J). Taken together, both ELISA and western blot results further verified the credibility of our proteomic analysis.

### Correlation analysis between potential serum biomarkers and clinical information

To further demonstrate the clinical utility of our serum proteomic result, we analyzed the correlation between potential serum biomarkers and clinical information. Compared to the healthy control group, pre-transplant patients displayed pancytopenia (reduced white blood cell (WBC), red blood cell (RBC) and platelet (PLT)), lower neutrophil (NEU), lymphocyte (LYM) and monocyte (MONO), lower liver synthesis function markers (cholesterol (CHO), albumin (ALB) and fibrinogen (FIB)), but higher gamma-glutamyl transferase (GGT), total bilirubin (TBIL) and prothrombin time (PT). AR and ITBL patients also displayed pancytopenia, and lower LYM, lower liver synthesis function markers (ALB and FIB), but higher ITBL and PT, compared to the healthy controls. AR patients were more prone to having worse liver function with higher liver enzyme markers (ALT, AST, and GGT). The clinical laboratory values listed above (Supplementary Table 5) are consistent with clinical routine and previously published articles. In the perioperative group, ACLY was positively correlated with CHO and ALB, and FGA and APOA1 were positively correlated with CHO, ALB, and FIB (Table 2). Serum ACLY and FGA levels were not correlated with any laboratory value in the AR and ITBL groups, which is likely due to the smaller sample size.

## DISCUSSION

Despite the standardization and advancements in liver transplantation in the last few decades, lack of prognostic markers remains a challenge [16]. Due to the shortage of donor organs globally, grafts from extended criteria donors, donation after death, and partial/suboptimal donors are used in transplantations. Their use has been associated with primary non-function (PNF), early allograft dysfunction (EAD) and severe biliary complications like ITBL [17]. The consistently rising incidence of ITBL is ascribed to the poor understanding of underlying mechanisms and the lack of effective diagnostic markers. Although immunosuppressants are widely used, AR is still the main obstacle for the prognosis after transplantation. The main reason is that it is difficult to detect AR early and balance suitable immune monitoring systems to guide individualized therapy. Although the pathophysiology of these complications is assumed to differ, ischemia-reperfusion injury has been identified as an important common risk factor [18]. Therefore, early diagnosis of postoperative complications is an important factor affecting mortality and prognosis in liver transplantation. Unfortunately, the sensitivity and specificity of the abnormal liver function test and ultrasound are low. At the same time, although puncture biopsy and cholangiography are the gold standards, they still face technical challenges, and are invasive and time-consuming. Early diagnosis of complications and appropriate intervention can lead to a better outcome. ESLD is a complex physical and psychological process, which is associated with profound protein profile changes. Detection of these protein expression changes during the perioperative period will help to better understand its underlying mechanism. It is always preferable to seek non-invasive, clinically useful biomarkers in this manner; biomarkers which reflect disease burden and could be applied for diagnosis of complications and monitoring of effectiveness after liver transplantation.

Proteomic approaches are types of techniques focusing on protein or peptide expression-level change and are widely used in the discovery of disease biomarkers [19]. MALDI-TOF MS is a valuable tool for the profiling of biological samples with relatively low abundance that is fast, highly sensitive, and has a high throughput [20]. In addition, the use of MALDI-TOF MS combined with magnetic beads is a new method that has been used to identify cancer and other diseases [21–25]. This method is also suitable for detection of serum protein biomarkers in liver transplant patients. However, it has some limitations. It is semi-quantitative to identify relative differences between samples for particular peak, and it requires complicated bioinformatic procedures for peak identification and data analysis.

In our study, three peptides were identified which showed the most significant changes in abundance between pre-transplant patients and healthy controls. Expression levels of these peptides changed significantly after removal of the failing liver, suggesting that they could be powerful indicators for monitoring the efficacy of transplant treatment. Furthermore, the two most significant peaks were selected as candidate biomarkers for AR and ITBL patients. These were further identified as the peptides ACLY, FGA, and APOA1. The same trend was verified *via* ELISA and western blot analysis in patient samples accordingly. We also found that ACLY was positively correlated with CHO and ALB, and FGA and APOA1 were positively correlated with CHO, ALB, and FIB in pre-transplant patients. Therefore, our data suggested these three peptides may play a vital role in understanding possible mechanisms, evaluating effectiveness, and early diagnosis of AR and ITBL.

ACLY is a cytosolic homotetrameric enzyme that catalyzes generation of acetyl-CoA from citrate and is involved in lipid biogenesis linked with glucose metabolism. Liver-specific ACLY deficiency protects mice from hepatic steatosis and dyslipidemia [26]. Moreover, increased ACLY expression has been recently reported in colon, lung, prostate, breast, and liver tumors by suppressing proliferation [27]. Inhibition of ACLY may be a promising therapeutic approach to dyslipidemia, atherosclerosis and cancer. Here, we found that ACLY expression was dysregulated in various groups. Changes in the impairment of liver synthesis function are common in pre-transplant, AR, and ITBL patients, which may partly due to a disorder of the glycolipid metabolism caused by decreased ACLY. This is the first report of the downregulation of ACLY in serum samples of pre-transplant, AR, and ITBL patients, suggesting ACLY could be a potential biomarker for effectiveness monitoring and complication diagnosis.

FGA encodes the alpha components of human FIB, which is a glycoprotein secreted by hepatocytes. FIB mainly affects the final step of the coagulation cascade, wound healing, inflammation, and angiogenesis. A number of studies have shown that elevated levels of plasma FIB are closely related to tumor progression in gastric cancer, lung cancer, colon cancer, and hepatocellular cancer [28–30]. In addition to tumor progression, FGA was also reported to be the acute phase protein with differential expression in response to injury and inflammation. Francis and Armstrong reported that dysfibrinogenemia did not appear to be related to the degree of liver function impairment, but might be associated with regeneration of hepatic tissue [31]. FGA has been described as a potential marker for AR following heart transplantation [32]. However, few studies have evaluated its value for liver transplantation. In our study, FGA was first identified to be a down-regulated protein in pre-transplant patients, while its high expression level indicated good prognosis for transplantation, which is likely due to the recovery of coagulation and tissue regeneration. We also found that FGA is down-regulated in AR and ITBL patients, suggesting that they could be potential serum biomarkers for patients who respond well to liver transplantation and for diagnosis of complications.

APOA1 is a principal protein component of high-density lipoprotein (HDL) produced by the liver. APOA1 is crucially involved in stimulating the ABCA1 transporter, which is part of the initial step of the reverse cholesterol transport and atheroprotective mechanisms [33]. APOA1 has been reported to be involved in many kinds of malignancies and Alzheimer’s disease [34]. Metabolic disorders are common clinical symptoms in various hepatic diseases. Li et al [35] found that serum levels of HDL (>0.93 mmol/L), APOA1 (>1.08 g/L) and LDL (≤2.62 mmol/L) among patients before liver transplantation were closely associated with postoperative survival in the univariate analysis. APOA1 is involved in regulating both lipid and energy metabolism, which may play important roles in liver regeneration *in vitro* and *in vivo* after transplantation [36, 37]. In the current study, the concentration of APOA1 in pre-transplant patients was significantly lower than that in healthy controls, but was elevated significantly after transplantation. Our findings suggest for the first time that APOA1 is a potential biomarker that can reflect disease burden and treatment effectiveness. There have been a limited number of studies focusing on AR diagnosis biomarkers for renal, heart and liver transplantation [12, 32, 38, 39]. To the best of our knowledge, this is the first study to identify potential biomarkers for perioperative, AR, and ITBL patients through MALDI-TOF MS in liver transplantation. Our results firstly revealed three potential serum biomarkers (ACLY, FGA, and APOA1) for monitoring effectiveness after liver transplantation, and two potential serum biomarkers (ACLY and FGA) for diagnosing AR and ITBL, validated by ELISA and correlation analysis with clinical data. All the identified proteins are hepatic synthesized and involved in biological processes such as glucolipid metabolism and the clotting cascade, which are both a reflect of liver synthesis function. In conclusion, serum ACLY, FGA, and APOA1 screening by high-throughput proteomics may be a promising, non-invasive, inexpensive and quick approach for predicting effectiveness and diagnosis of complications after liver transplantation.

The study had several limitations. Firstly, the sample size in this study was relatively low due to difficulty in obtaining serum samples from the study population. The proposed biomarkers need to be validated in a bigger, more diverse, and independent clinical cohort. Secondly, serums were acquired only at a single point of time, and lacked a time gradient. Thirdly, the proteomics is a semi-quantitative approach to identify relative differences in peaks among samples; moreover, in clinical practice the potential biomarkers should be used in combination with other imaging and laboratory examination. Finally, since ACLY, FGA, and APOA1 are common biomarkers for prognosis and diagnosis of AR and ITBL, they might also reflect the recovery of liver function and nonspecific reactions to diseases, rather than relative specific indicators. Therefore, further studies are required to investigate biological function *in vivo* and *in vitro* to detail the underlying molecular mechanisms.

## MATERIALS AND METHODS

### Patients and samples

All serum samples and clinical information were collected after obtaining patients consent in accordance with the declaration of Helsinki for patients receiving standard orthotopic liver transplantation from January 2016 to January 2017. The study was approved by the ethics committee of the First Affiliated Hospital of Xi’an Jiaotong University. Each patient was followed-up after discharge at least every 2 weeks in the first year and every 2 months in the second year. 20 serum samples were obtained from 10 pairs of patients before and 14 days after transplantation. In addition, we screened AR patients (n = 10): patients newly diagnosed as AR with Banff criteria and ITBL patients (n = 9): patients newly diagnosed as ITBL using endoscopic retrograde cholangiography (ERC). All samples were obtained within 48 hours after the onset of AR or ITBL, and then the initial treatment was carried out. All the patients (n = 10) recovered well after transplantation, which we call excellent patients. Blood samples of patients without any complications were collected 14 days after surgery and followed up at least 2 years.. Blood samples from 10 healthy donors who were matched for age and gender without any evidence of diseases were also collected as controls.

The serum samples were collected in 10 ml vacuum tubes without anticoagulants and were kept at 4 °C for 1 h, then centrifuged at 3,000g for 15 min at 4 °C. The serum samples were distributed in 500 μl aliquots and stored at −80 °C until use.

### MALDI-TOF MS analysis

Serum samples were separated using MB-WCX chromatography (Bruker Daltonics, Bremen, Germany) according to the manufacturer’s protocol. With the magnet lowered, 5 μl serum samples were diluted in 10 μl binding solution in a standard thin well PCR tube, added to 10 μl of MB-WCX beads and mixed by pipetting up and down. After incubated for 5 min at room temperature, the tube was placed into the magnetic separator and the beads were collected on the wall of the tube until the supernatant was clear (~1 min). The supernatant was then removed carefully and the magnetic beads were washed three times with washing buffer. Next, we eluted the peptide fraction from the magnetic beads with 5 μl of elution solution and 5 μl stabilization buffer. Finally, we spotted 1 μl eluted peptide and 1μl alpha-cyano-4-hydroxycinnamic acid (Bruker Daltonics) in 50% acetonitrile onto MALDI-TOF MS targets, and added 0.5% trifluoroacetic acid twice to the MALDI AnchorChip surface. All samples were spotted in triplicate to evaluate reproducibility.

### ClinProTools analysis

All targets were analyzed using a calibrated Autoflex III MALDI-TOF MS (Bruker) immediately, with an optimized protocol of FlexControl software (Version 3.0, Bruker). Mass calibration was executed with a standard calibration mixture of peptides and proteins (mass range, 1,000-10,000 Da). All tests were performed in a blinded manner, including the serum analysis of different groups. All serum data were analyzed by Flex analysis software (Version 3.0; Bruker) and recognition of peptide patterns was performed using ClinProTools software (Version 2.2; Bruker), including spectra pretreatment, peak detection and peak calculation operation.

### Peptide identification

The peptides were analyzed by liquid chromatography-mass spectrometry (LC-MS), which integrate Nano Acquity UPLC (Waters, USA) with LTQ Orbitrap XL mass spectrometer (Thermo Fisher Scientific, USA). 20 μl samples were trapped by C18 column (Symmetry^®^C18, 3 μm, 0.10×20 mm, nanoAcquity™Column) at 600 nl/min for 3 min and then loaded on analytical column (Symmetry^®^C18, 1.9 μm, 0.15×120 mm, nanoAcquity™ Column) at 400 nl/min for 60 min. Mobile phase A was a solution of 5% acetonitrile and 0.1% formic acid, and mobile phase B was a solution of 95% acetonitrile and 0.1% formic acid, and the column temperature was maintained at 35°C. The LTQ-Orbitrap XL mass spectrometer was operated in the data-dependent mode to switch automatically between MS and MS/MS acquisition. Survey full scan MS spectra with 2 microscans (m/z 300–1400) were acquired with the Q Exactive with a mass resolution of 70,000 at m/z 300, followed by 10 sequential LTQ-MS/MS scans. Dynamic exclusion was used with 2 repeat counts, with 18s repeat duration and 80s exclusion duration. For MS/MS, precursor ions were activated with 25% normalized collision energy at the default activation q of 0.25. The mass spectra were searched against the Uniprot database (https://www.uniprot.org/) using Mascot software (Version 1.5.2.8). To reduce false positives, a decoy database containing reverse protein sequences was added to this database. The search parameters were as follows: no enzyme, the variable modification was oxidation of methionine, peptide tolerance of 15 ppm, fragment mass tolerance of 20 mmu. Positive protein identification was confirmed with Peptide FDR ≤ 0.01.

### Bioinformatics analysis

Functional enrichment and interaction network analysis tool (Funrich, http://www.funrich.org) was used to analysis cellular components, molecular functions, biological processes and biological pathway of the identified peptides. The interaction network of the differentially expressed proteins was built automatically by the STRING (Search Tool for the Retrieval of Interacting Genes/Proteins) system (http://string-db.org) with default settings.

### ELISA

All serum samples were analyzed blindly in triplicate and the concentrations of ACLY (Shanghai Hengyuan, H-12925), FGA (Shanghai Hengyuan Biotech, H-12843) and APOA1 (Shanghai Hengyuan, H-11508) were measured according to the manufacturer’s instructions respectively.

### Western blot

Western blot was used to quantify the protein expression of ACLY, FGA and APOA1. 10 μg of protein from serum samples were separated on 10% SDS-PAGE, transferred to PVDF membranes, and incubated with 1:1000 diluted primary antibodies against ACLY (GeneTex, GTX60666, Monoclonal), FGA (Proteintech Group, 20645-1-AP) and APOA1 (Proteintech Group, 14427-1-AP).

### Statistical analysis

SPSS 25.0 statistical software and GraphPad Prism Version 6.0 (GraphPad Software, La Jolla, CA, USA) were used for statistical analysis. Data are shown as mean ± standard deviation and P<0.05 was considered statistically significant. Differences were evaluated using Student’s t test for continuous parametric data, Wilcoxon test for continuous nonparametric data, and Pearson’s chi-squared test for noncontinuous data. Pearson correlation analysis was used to determine the correlation between expression of serum biomarkers and clinical information.

## Supplementary Material

Supplementary Figure 1

Supplementary Tables
