# Supplementary material for: Serum proteomic predicts effectiveness and reveals potential biomarkers for complications in liver transplant patients
Source: Aging (Albany NY). 2020 Jun 12;12(12):12119–41. doi: 10.18632/aging.103381 (PMC7343480; doi:10.18632/aging.103381)
Supplement: Supplementary Figure 1 [file aging-12-103381-s001..pdf]

SUPPLEMENTARY FIGURE

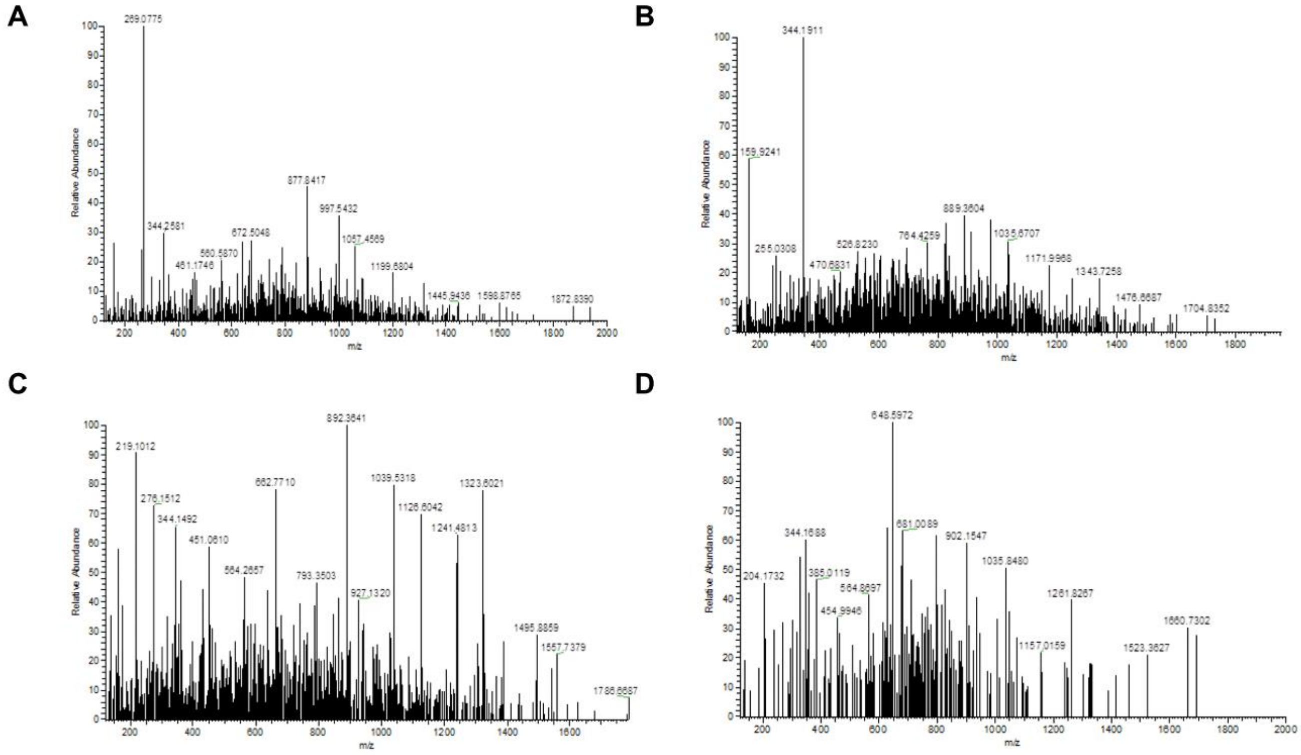

Supplementary Figure 1. MS/MS spectrometry fragment map. (**A**) Peak 1, m/z: 4100.81. (**B**) Peak 2, m/z: 1949.9. (**C**) Peak 3, m/z: 2666.86. (**D**) Peak 4, m/z: 2087.71.
