# Supplementary material for: Serum proteomic predicts effectiveness and reveals potential biomarkers for complications in liver transplant patients
Source: Aging (Albany NY). 2020 Jun 12;12(12):12119–41. doi: 10.18632/aging.103381 (PMC7343480; doi:10.18632/aging.103381)
Supplement: Supplementary Tables [file aging-12-103381-s002..pdf]

SUPPLEMENTARY TABLES

Supplementary Table 1. Demographics of all subjects enroll in this study.

Variable	ARs N=10	ITBLs N=9	Transplant excellents N=10
**Donor**			
Age, years	43.14±13.93	55.75±10.01	42.54±17.47
Sex, M/F	9/0	7/2	9/1
Body mass index, kg/m^2	22.72±3.63	24.77±1.60	20.46±3.99
**Etiology**			
cerebral hemorrhage	1	2	3
craniocerebral injury	8	7	6
others	1	0	1
**Recipient**			
**Preoperative**			
Age, years	46.57±8.75	52.00±9.60	47.88±8.49
Sex, M/F	8/2	7/2	7/3
Body mass index, kg/m^2	22.06±2.03	22.09±3.90	20.80±4.94
**Etiology**			
Liver cirrhosis	8	6	8
Hepatitis B virus related	4	4	4
Hepatitis C virus related	1	0	2
Alcohol related	0	0	1
Others	3	2	1
Hepatocellular carcinoma	1	1	1
Fulminant Hepatic Failure	1	2	1
**Child-plug score**			
B	4	4	4
C	6	5	6
MELD score	18.71±5.39	19.00±6.10	20.88±6.60
**ABO-compatible**			
Yes	9	9	9
No	1	0	1
**Intraoperative**			
Cold ischemia time, hours	6.00±1.41	6.00±1.26	6.50±2.29
Warm ischemia time, minutes	14.57±7.28	10.60±1.74	9.63±0.86
Blood loss, ml/kg	22.28±8.65	25.22±11.24	28.69±12.28
Operation time, hours	5.86±0.52	6.26±1.14	6.59±1.10

RBC transfusion, units	8.29±3.28	10.00±6.69	11.00±3.74
Plasma transfusion, ml	1542.86±905.31	1320.45±640.27	1650.00±396.86
Anhepatic phase, minutes	48.71±10.50	57.60±7.28*	45.88±8.43
**Postoperative**			
Banff score, RAI	9.00±0.00	---	---
Immunosuppression			
Acrolimus+MMF+steroids	6	5	7
Cyclosporin+MMF+steroids	4	4	3
Mean immunosuppression concentration in the first week			
Low	6	2	1
Normal	4	5	4
High	0	2	5
Biliary drainage			
Yes	4	2	4
No	6	7	6
Biliary infection			
Yes	8	9	8
No	2	0	2
Mean velocity of hepatic arterial in the first week, cm/s	53.34±13.45	51.86±15.92	55.68±15.64
CMV infection			
Yes	2	0	0
No	8	9	10
Diagnosis postoperatively, days	32.14±26.21	78.00±35.20	---
Treatment	corticosteroids+ adjust/increased immunosuppression	ERC + repeated biliary stent	---
Prognosis	survival	survival	survival

Comparison of general information between AR, ITBL and transplant excellent patients.

Supplementary Table 2. Peaks showing significant differences in abundance across samples for perioperative group.

Peak	m/z	P value	Controls (N=10)	Post-transplants (N=10)	Pre-transplants (N=10)
**1**	**1949.82**	0.000126	35.35±10.79	18.03±12.37	3.16±1.02
**2**	**4100.54**	0.000321	8.31±2.18	5.46±2.60	2.05±1.66
**3**	**2666.86**	0.000605	29.98±11.64	19.38±8.39	11.81±7.78
**4**	**4292.26**	0.000698	4.13±1.33	1.72±1.19	1.04±0.40
**5**	**3964.84**	0.000833	9.69±3.95	4.52±2.92	1.58±0.43
6	2087.59	0.000821	8.07±2.63	6.04±3.57	2.48±0.64
7	4081.73	0.00267	2.18±0.35	1.74±0.44	1.11±0.40
8	2890.2	0.00338	4.41±1.22	4.70±2.51	1.91±0.71
9	4276.49	0.00104	2.91±1.10	1.44±0.76	0.78±0.22
10	4219.58	0.00321	20.64±7.53	12.74±7.56	4.66±5.13
11	2215.65	0.00042	8.16±2.84	6.34±2.83	1.95±0.44
12	4062.93	0.00419	6.23±1.87	5.87±3.69	2.79±1.21
13	2869.39	0.00924	9.41±4.35	9.73±6.92	3.43±1.65
14	1992.86	0.00924	6.07±2.86	3.32±2.17	1.89±0.66
15	1549.95	0.00961	13.65±5.74	7.97±3.88	4.97±2.46
16	4203.38	0.00775	3.34±0.96	2.05±0.71	1.76±0.82

Supplementary Table 3. Peaks showing significant differences in abundance across samples for AR group.

Peak	m/z	P value	Controls (N=10)	Transplant excellents (N=10)	ARs (N=10)
**1**	**1950.06**	0.0000219	34.55±10.53	12.00±4.34	2.64±0.87
**2**	**2087.9**	0.000906	7.44±2.02	4.52±1.38	2.70±1.12
3	4292.53	0.0000927	4.24±1.22	1.01±0.22	1.00±0.30
4	3965.22	0.000802	7.41±3.43	5.47±2.05	2.00±0.87
5	2216	0.000908	8.03±2.91	4.11±1.33	3.66±0.71
6	3981.27	0.00106	2.21±0.48	1.82±0.63	1.16±0.19
7	1325.04	0.0011	5.44±1.81	2.52±0.75	1.91±0.38
8	4276.88	0.0013	3.05±0.98	1.07±0.31	1.19±0.68
9	6818	0.0016	0.30±0.09	0.50±0.14	0.90±0.35
10	2278.14	0.00172	10.91±6.45	4.01±1.78	1.58±0.37
11	808.27	0.00094	6.68±1.96	8.66±1.65	13.53±3.51
12	1659.42	0.00414	6.23±2.68	2.14±0.85	1.85±0.51
13	883.49	0.00424	1.90±0.65	4.76±1.86	7.92±7.11
14	2679.57	0.00424	3.01±0.73	6.14±1.84	4.81±3.64
15	4485.44	0.00556	1.89±0.77	0.76±0.16	0.71±0.11
16	4655.21	0.0067	3.19±1.57	0.87±0.29	1.10±0.46
17	925.61	0.0074	1.68±1.04	3.83±1.49	8.66±7.52
18	5918.28	0.0074	8.94±4.30	4.97±2.89	2.28±2.31
19	5954.23	0.0074	1.05±0.22	0.96±0.49	0.61±0.23
20	7783.57	0.00742	3.71±2.52	0.53±0.21	1.14±0.71
21	3892.45	0.00786	2.81±1.37	1.01±0.32	1.42±0.48
22	899.4	0.00886	3.69±2.08	7.55±2.53	24.76±27.88
23	4220.05	0.00711	22.76±7.18	12.94±5.68	8.60±10.29

Supplementary Table 4. Peaks showing significant differences in abundance across samples for ITBL group.

Peak	m/z	P value	Controls (N=10)	Transplant excellents (N=10)	ITBLs (N=9)
**1**	**2087.92**	0.000339	6.94±2.08	4.16±1.35	2.61±0.75
**2**	**1949.99**	0.000232	30.07±12.69	11.45±4.36	4.87±2.49
3	2215.91	0.000705	8.06±2.57	2.98±2.76	1.99±0.85
4	2997.9	0.00108	3.42±0.68	10.37±6.84	2.11±0.63
5	3965	0.00108	9.16±3.95	3.93±1.91	2.85±1.91
6	4655.4	0.00108	3.08±1.44	0.81±0.27	1.56±0.49
7	4276.46	0.00108	2.85±0.95	1.05±0.28	1.33±0.55
8	1659.29	0.00114	6.54±2.61	2.13±0.74	2.20±0.79
9	4292.4	0.00114	3.73±1.56	0.99±0.25	1.46±0.55
10	1992.76	0.00151	6.42±2.70	2.13±0.82	2.09±0.83
11	7782.77	0.00291	3.93±2.35	0.57±0.29	2.24±1.45
12	2697.17	0.00291	2.96±0.71	5.84±1.78	4.07±2.45
13	4485.36	0.00291	1.84±0.81	0.72±0.16	0.88±017
14	3892.21	0.00351	2.85±1.38	1.00±0.32	2.01±0.83
15	1436.45	0.00386	6.24±2.39	2.77±0.94	3.18±0.98
16	9311.1	0.00422	2.84±1.65	0.55±0.24	1.22±0.76
17	879.56	0.00557	4.83±2.16	4.81±1.93	2.95±0.39
18	2098.23	0.00795	3.48±1.09	7.79±3.34	7.06±5.48

Supplementary Table 5. Clinicopathological features of all subjects enroll in this study.

Variable	Controls	Transplant excellents	Pre-transplants	ITBLs	ARs
	N=10	N=10	N=10	N=9	N=10
**Hematological parameters**					
WBC, x10^9/L	6.52±1.38	6.71±2.63	3.04±0.75***	4.40±1.71**	3.22±1.73***
RBC, x10^{12}/L	4.92±0.67	3.67±0.37**	3.44±0.84***	3.05±0.38***	3.42±0.55***
PLT, x10^9/L	219.40±49.82	225.17±116.16	59.70±61.94***	63.89±14.64***	45.40±18.50***
NEU, x10^9/L	4.51±0.76	5.42±2.00	1.89±0.57***	3.35±1.59	2.27±1.58***
LYM, x10^9/L	2.44±0.47	0.72±0.42***	0.86±0.38***	0.53±0.17***	0.47±0.44***
MONO, x10^9/L	0.41±0.08	0.45±0.19	0.21±0.10***	0.42±0.20	0.22±0.14**
**Liver function markers**					
AST, U/L	20.09±6.69	18.33±8.19	143.30±318.00	101.44±187.97	168.30±208.03*
ALT, U/L	22.60±11.27	42.5±20.18	98.8±21.46	68.44±114.55	131.5±138.54*
*GGT, U/L	24.20±10.29	63.67±24.23***	45.40±29.73*	51.42±33.31*	201.90±204.21*
CHO, mmol/L	3.99±0.94	4.29±0.43	3.05±0.86*	2.57±1.00**	3.14±1.22
ALB, g/L	46.30±4.35	38.07±3.89**	33.83±5.22***	31.18±4.31***	34.67±5.65***
TBIL, umol/L	8.94±3.31	24.03±12.46**	70.44±66.17**	121.65±106.89**	93.54±76.56**
**Coagulation function makers**					
PT, s	12.40±0.68	13.45±1.01*	21.31±5.72***	18.13±3.70***	17.51±3.06***
FIB, g/L	3.03±0.56	3.26±0.47	1.42±0.51***	1.92±0.36***	1.73±0.47***

(*** indicates P<0.001, ** indicates P<0.01, * indicates P<0.05)
